# Redefining Aortic Valve Replacement: The Transition from Open Surgery to Transcatheter Innovation

**DOI:** 10.3390/jcm14196761

**Published:** 2025-09-24

**Authors:** Frances Lodge, Fady Soliman, Viswajit Kandula, Marco Tagliafierro, Luigi Pirelli

**Affiliations:** Division of Cardiothoracic Surgery, Department of Surgery, Columbia University Irving Medical Center, New York, NY 10032, USA; fl2638@cumc.columbia.edu (F.L.); fks7001@nyp.org (F.S.); vk2509@cumc.columbia.edu (V.K.); mt3672@cumc.columbia.edu (M.T.)

**Keywords:** aortic valve replacement (AVR), surgical aortic valve replacement (SAVR), Transcatheter aortic valve replacement (TAVR), bioprosthetic valves, mechanical valves, structural valve degeneration (SVD), valve-in-valve (ViV), aortic stenosis

## Abstract

Aortic valve replacement (AVR) has evolved dramatically, transitioning from open surgery to minimally invasive and transcatheter approaches. This review examines the historical and technological advancements in AVR, focusing on the evolution of mechanical and bioprosthetic valves, valve-sparing techniques, and the Ross procedure. Mechanical valves offer superior durability but require lifelong anticoagulation, while bioprosthetic valves avoid this need at the expense of long-term durability. Transcatheter aortic valve replacement (TAVR), originally reserved for high-risk patients, is now used across all risk profiles due to growing evidence of safety and efficacy from major trials like PARTNER and Core Valve. Despite its benefits, TAVR presents some challenges, including paravalvular leak, pacemaker implantation, and uncertain long-term durability, especially in younger patients. Valve-in-valve techniques, novel valve designs, and anti-calcification treatments offer promising solutions. Looking forward, the integration of artificial intelligence and personalized procedural planning will play a key role in optimizing outcomes. As AVR technology advances, careful patient selection and a multidisciplinary approach remain essential to guiding individualized treatment decisions.

## 1. Introduction

The field of cardiothoracic surgery has undergone remarkable transformation over the past several decades. Aortic valve replacement (AVR), in particular, has seen exponential growth and innovation. The advent of cardiopulmonary bypass in 1953 laid the groundwork for modern cardiac surgery. Just eight years later, the first surgical aortic valve replacement was performed in 1961 with a Starr-Edwards valve [1]. Since then, the management of AVR has evolved into a highly specialized discipline with multiple standard approaches tailored to individual patient needs. The pursuit of less invasive strategies has driven a paradigm shift—from traditional open surgical valve replacements to minimally invasive options, and more recently, to transcatheter valve interventions. This review explores the historical and technological evolution of aortic valve therapies, tracing the progression from early surgical valve replacement with mechanical and biological prosthesis to the advent of aortic valve repair techniques and the transformative impact of transcatheter valve replacement (TAVR).

## 2. Surgical Aortic Valve Replacement (SAVR)

Aortic valve disease, which can manifest as stenosis, regurgitation, or mixed pathology, is a common condition with significant prevalence in the Western world [2]. Traditionally, the gold standard therapy for aortic valve disease has been valve replacement. Two main types of prosthetic valves exist: mechanical valves, which offer superior durability but necessitate lifelong anticoagulation, and bioprosthetic valves, typically derived from bovine or porcine pericardium, which require only antiplatelet agents but have limited durability. Despite the innovations in longevity and hemodynamic profiles of newer generation surgical valves, SAVR techniques still fundamentally rely on cardiopulmonary bypass and aortic cross-clamping.

### 2.1. Mechanical SAVR

**Figure 1:**
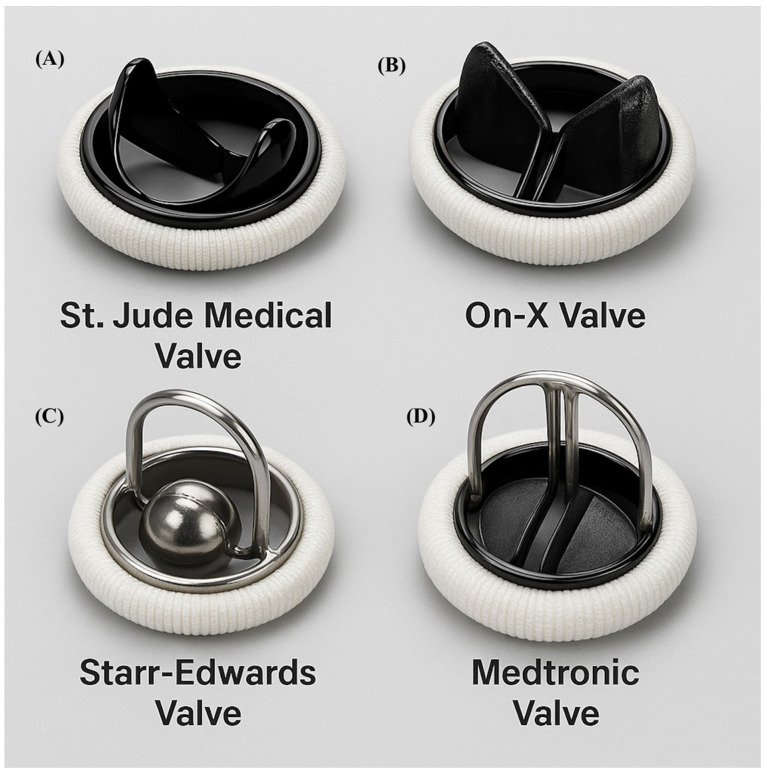
Mechanical valve types. (**A**). Saint Jude Medical. (**B**). On-X. (**C**). Starr-Edwards (**D**). Medtronic.

Mechanical valves, constructed from durable materials such as pyrolytic carbon and titanium, are highly resistant to structural deterioration. However, their thrombogenic surfaces require lifelong anticoagulation, typically with a vitamin K antagonist. There are three types of mechanical valves: caged ball valves, non-tilting disc valves, and bileaflet valves (Figure 1).

Caged-Ball: The Starr-Edwards valve, introduced in 1961, was the first clinically used prosthesis (Figure 1C). It featured a silicone ball housed within a metal cage [1]. Despite historical significance, valves were discontinued in 2007 due to high thromboembolic risk and suboptimal hemodynamics [3].

Tilting Disc: Introduced in 1969 by Bjork and Shiley, these valves employed single tilting discs to regulate unidirectional flow [3,4]. Although improved upon earlier designs, they have been supplanted by bileaflet valves due to better hemodynamic profiles and lower complication rates.

Bileaflet: First introduced in 1977 (e.g., St. Jude Medical valve), the bileaflet design remains the most commonly used mechanical prosthesis today (Figure 1A). They feature two semicircular leaflets housed within a sewing ring, offering excellent hemodynamic and durability [3,5].

#### 2.1.1. The On-X Valve

Introduced in 1996, the On-X valve represents a modern iteration of mechanical valve design (Figure 1B). FDA approval for aortic implantation followed the PROACT trial, which demonstrated that patients with On-X valves could be maintained on a lower INR target of 1.5–2.0, without increased thromboembolic risk [6,7,8]. Long-term follow-up data support the valve’s excellent durability and reduced bleeding complications, making it a preferred option for many patients opting for mechanical AVR.

#### 2.1.2. Challenges/Management

Despite their durability and superb hemodynamic profiles, mechanical valves carry risks such as thrombosis, pannus formation, and bleeding associated with anticoagulation. A systematic review reported an overall valve-related complication rate of 0.7–3.5% per patient year for mechanical aortic prostheses, with specific risks including thromboembolism (~1.0% per patient year), major bleeding (~0.5% per patient year), endocarditis (<0.5% per patient year), and non-structural dysfunction such as paravalvular leak or pannus formation (0.4–1.2% per patient year) [9]. Mechanical valve thrombosis (MVT) can be a life-threatening complication and may occur in patients that are not compliant with anticoagulation regimens. Management strategies range from i.v. anticoagulation with heparin, to thrombolysis, to surgical re-intervention, depending on severity [10].

### 2.2. Bioprosthetic SAVR

**Figure 2:**
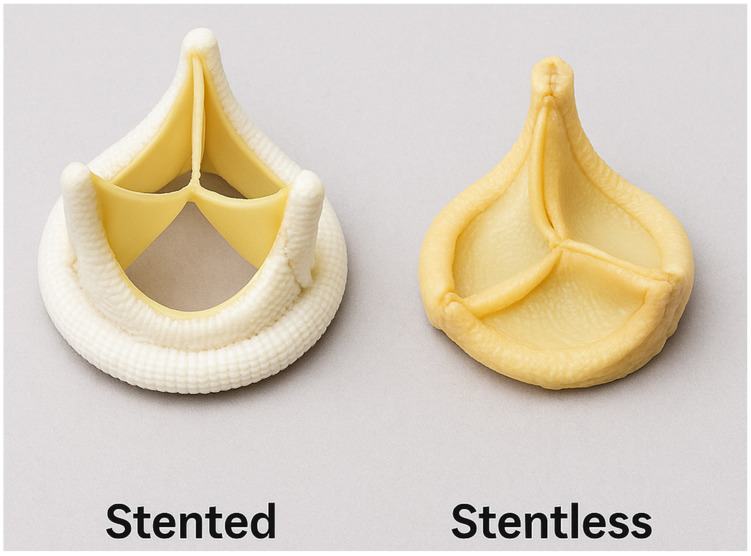
Bioprosthetic valve types: stented vs. stentless.

Bioprosthetic valves are composed of xenograft tissue—typically bovine or porcine pericardium, treated with anti-calcification processing and immunogenic treatments to facilitate long-term durability [11,12,13]. There are two types of bioprosthetic valves: stent valves and stentless valves (Figure 2).

Stent valve: These valves consisted of xenograft tissue mounted on a synthetic frame to mimic native valve anatomy with a sewing ring in place to facilitate easier implantation and standardized sizing. The first aortic bioprosthetic stent valve was implanted in 1965 by Alain Carpentier using a porcine valve manufactured by Edwards Laboratories (now Edwards Lifesciences).

Stentless valve: These valves lack a supporting frame, allowing for a more physiological flow profile and potentially superior hemodynamic performance, especially in patients with a small aortic annulus. They mimic the aortic root geometry more closely and offer improved effective orifice area and lower transvalvular gradients. The concept of stentless valve design was first introduced in the 1960s, representing an early attempt to preserve natural flow dynamics in valve replacement surgery [14,15].

These bioprosthetic valves are now favored given their excellent hemodynamic profiles and ability to avoid lifelong anticoagulation, making them appealing choices for elderly populations or those with contraindications to blood thinners. Reflecting this shift, the proportion of bioprosthetic surgical aortic valve replacements has increased from 11.5% to 51.6% from 1996 to 2013 [16]. Stentless valves, while offering superior hemodynamic performance, have declined in popularity due to more technically challenging implantation, longer operative times, and variable long-term outcomes. Consequently, stented bioprosthetic valves have become the preferred choice for most surgical candidates requiring a tissue valve, balancing ease of use, favorable hemodynamics, and patient safety [17].

#### 2.2.1. Ross Procedure

An alternative biologic approach to aortic valve replacement is the Ross procedure. This procedure involves replacing a diseased aortic valve with the patient’s own pulmonary valve (autograft), while a pulmonary homograft is used to replace the excised pulmonary valve. Originally described by Dr. Donald Ross in 1967, this technique offers excellent hemodynamics, avoids lifelong anticoagulation, and shows favorable long-term outcomes in young patients [18,19,20]. However, it is technically complex and involves the replacement of two valves, introducing potential long-term risks to the right ventricular outflow tract [21].

#### 2.2.2. Challenges/Management

Despite their favorable hemodynamic profile and reduced thrombogenicity, bioprosthetic valves are limited by their finite durability, with structural valve degeneration (SVD) typically occurring within 10–15 years post-implantation [12]. SVD manifests as progressive stenosis, regurgitation, or both, leading to classic valvular symptoms such as exertional dyspnea, angina, presyncope, and fatigue. Reintervention becomes inevitable upon valve failure, posing a significant concern for younger patients who face the cumulative risk of multiple reoperations over their lifetime [22]. Of note, calcific degeneration remains the primary mechanism by which bioprosthetic valves fail. This process leads to leaflet stiffening, reduced cusp mobility, and valvular dysfunction. Accelerated degeneration is associated with several risk factors, including younger age, chronic kidney disease, hypercalcemia, and increased mechanical stress. Notably, younger patients are more prone to rapid calcification due to higher metabolic activity and calcium turnover, which results in decreased valve durability [23,24].

### 2.3. Advances in Tissue Valve Durability

**Figure 3:**
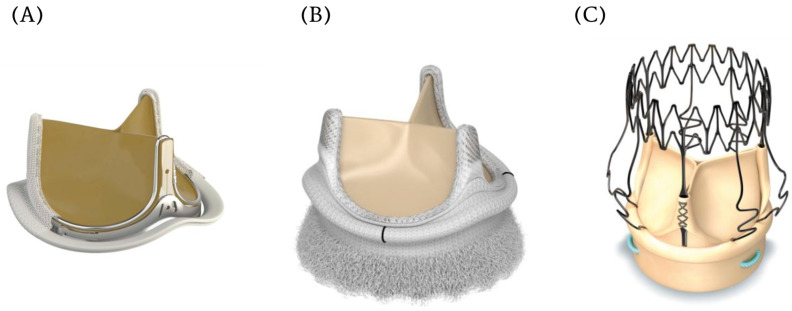
Contemporary innovations in bioprosthetic aortic valves. (**A**). Edwards Inspiris Resilia valve (**B**). Edwards Intuity Elite aortic valve (**C**). Perceval Sutureless aortic valve.

Multiple studies are currently underway evaluating strategies to improve valves durability and limit structural valve deterioration. One of the bioprosthetic valves that is drawing particular attention is the Edwards Inspiris Resilia valve (Figure 3A). Made of bovine pericardial tissue, this valve utilizes a novel tissue preservation technology, incorporating stable functional group capping, to reduce leaflet calcifications and glycerolization to allow for dry storage, thereby easing handling of the valve and enhancing valve durability [25]. In vitro studies suggest that the durability of Resilia valve may be up to 25 years, far exceeding any available bioprosthetic valve currently on the market [26]. The 7-year results of the COMMENCE TRIAL, a prospective non-randomized multicenter study evaluating the safety and efficacy of the Resilia Valve, demonstrated 99.3% freedom of structural valve degeneration (SVD) and excellent valve hemodynamics at 7 years [27]. Furthermore, the expandable stent frame of the Resilia valve, “V-Fit Technology”, available in 19–25 mm size valves, will expand valve–valve options allowing the implantation of a larger size TAVR. While promising, large multicenter randomized control trials with long follow-up times have yet to evaluate the long-term efficacy of Resilia valve and true advantage over conventional bioprosthetic valves.

#### 2.3.1. Minimally Invasive AVR

Minimally invasive approaches to aortic valve replacement (mini-AVR) were developed to minimize surgical trauma, enhance recovery, and reduce the morbidity associated with conventional surgical aortic valve replacement (SAVR). While following the same fundamental principles as SAVR performed via a traditional sternotomy, mini-AVR is performed through smaller incisions, either partial hemi-sternotomy or right anterior thoracotomy. Multiple studies have demonstrated decreased postoperative pain, lower operative mortality, less bleeding and transfusion rates, shorter hospital stays and subsequently enhanced patient recovery [28,29,30,31]. Despite its proven benefits, there remain major barriers to the adoption of mini-AVR, namely the added case complexity and procedure time, as well as the need for surgeons’ experience, which have curtailed the widespread availability of this procedure, currently limited to only few centers of excellence.

#### 2.3.2. Rapid Deployment Sutureless AVR (Figure 3B,C)

To facilitate the adoption of mini-AVR and making the procedure technically easier and faster, there has been increasing interest in rapid deployment sutureless (RDS) bioprosthetic valves. These valves utilize a circumferentially expandable frame to seat and anchor the valve within aortic annulus, without the need for suture placement. The Perceval and Intuity valves are amongst the most commonly used RDS devices. While their benefits include ease of placement in narrow operative fields, and shorter cross-clamp and cardiopulmonary bypass times, some studies show similar operative and long-term mortality despite a higher risk of permanent pacemaker (PPM) implantation [32]. It is worth noting also that the use of RDS bioprosthetic valves is increasingly controversial, in light of new long-term studies demonstrating a trend towards increased SVD and lower valve durability [33].

### 2.4. Transcatheter Aortic Valve Replacement

**Figure 4:**
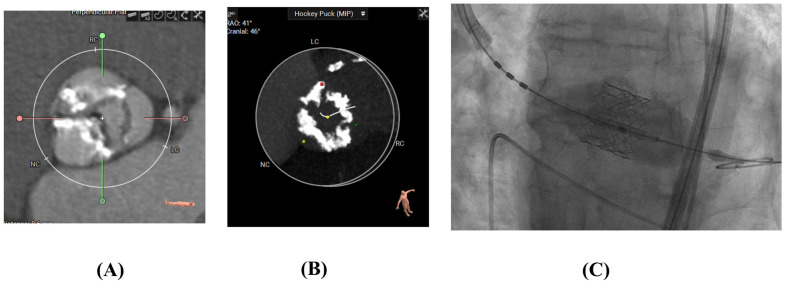
(**A**,**B**) CT scans of calcified aortic valves pre-TAVR (**C**). Edwards-Sapien TAVR Valve Deployed.

Transcatheter aortic valve replacement (TAVR) is a minimally invasive procedure developed as an alternative to SAVR, particularly for patients with symptomatic severe aortic stenosis. Rather than excising the native valve leaflets, TAVR involves delivering a bioprosthetic valve via a catheter, most commonly through transfemoral arterial access, although alternative access routes (e.g., transapical, transaortic, trans-carotid) may be used when necessary (Figure 4). The prosthetic valve—typically mounted on a balloon-expandable or self-expanding stent frame—is positioned within the diseased calcified native aortic valve. Upon deployment, the native leaflets are displaced outward by expanding the stent frame and anchoring the new valve in the native annulus, allowing it to assume the function of the native valve. This technique obviates the need for sternotomy, thoracotomy, or cardiopulmonary bypass. Since its first human implantation in 2002, TAVR has undergone rapid technological evolution and is now used across a broad spectrum of surgical risk profiles, including intermediate- and low-risk patients, with ongoing studies exploring its application in younger populations with less severe aortic stenosis and even aortic regurgitation.

#### 2.4.1. History

The development of TAVR represents one of the most transformative advances in structural heart disease. The concept emerged from two innovations: Alain Cribier’s balloon aortic valvuloplasty (BAV) in the late 1980s for inoperable patients with severe stenosis, and Henning Andersen’s early 1990s proposal of a balloon-expandable valve mounted on a stent for percutaneous delivery. This conceptual groundwork culminated in the first successful TAVR, which was performed by Dr. Cribier in April 2002 at the Charles Nicolle University Hospital in Rouen, France. The patient, a 57-year-old man with critical aortic stenosis, cardiogenic shock, and prohibitive surgical risk, underwent transseptal valve implantation after a failed BAV attempt. The immediate hemodynamic improvement confirmed the viability of percutaneous AVR, marking the start of a new era in cardiac intervention [34].

Following this successful procedure, Percutaneous Valve Technologies (PVT)—the company co-founded to develop early TAVR prototypes—was acquired by Edwards Lifesciences in 2004. This acquisition fueled iterative advancement in valve design, delivery systems, and access techniques, ultimately culminating in the SAPIEN valve platform.

The placement of AoRTic TraNscathetER valve (PARTNER) trials were pivotal in defining the clinical role of TAVR using balloon-expandable valves. In 2011, based on results from PARTNER 1B, the U.S. Food and Drug Administration (FDA) granted approval for TAVR in patients with severe symptomatic aortic stenosis (AS) at prohibitive operative risk [35]. In 2012, this approval was extended to high-risk patients. By 2015, TAVR was approved for use in valve-in-valve procedures for failed surgical bioprostheses.

**Figure 5:**
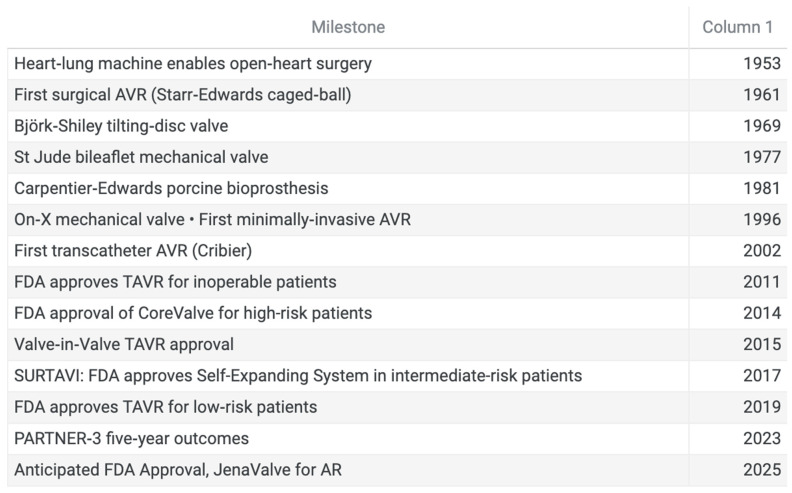
Timeline of significant milestones in aortic valve surgery.

In parallel, the CoreValve U.S. Pivotal Trial demonstrated the safety and efficacy of self-expanding TAVR devices in high-risk patients, showing significantly lower all-cause mortality at one year compared to surgery, leading to FDA approval of the CoreValve system in 2014 [36]. The SURTAVI trial later expanded indications to intermediate-risk patients, confirming non-inferiority of self-expanding TAVR versus SAVR in this population [37]. The PARTNER 2 trial supported approval for intermediate-risk patients in 2016, and by 2019, the PARTNER 3 trial established the non-inferiority of TAVR versus SAVR in low-risk patients [38]. More recently, in 2023, the 5-year follow-up of the PARNTER-3 trial was published, confirming that there was no statistically significant difference in outcomes between TAVR and SAVR in low-risk patients with severe, symptomatic aortic stenosis, reinforcing TAVR as an upfront option for management of AS across all surgical risk categories [39] (Figure 5).

#### 2.4.2. TAVR Valve Systems

Since its introduction in 2002, TAVR technology has undergone several rapid and iterative changes. Manufacturers have developed up to five generations of valve platforms, each one improving upon key design principles such as valve durability, deployment precision, paravalvular leak, pacemaker rate, etc. Today, the most commonly studied and utilized TAVR valve systems include the Edwards SAPIEN 3/Ultra, Medtronic Evolut PRO/PRO+, and Abbott Navitor.

#### 2.4.3. Edwards SAPIEN

The Edwards SAPIEN valve is a balloon-expandable intra-annular transcatheter heart valve and is currently the leading balloon-expandable valve platform. These valves are made from bovine pericardial tissue, which are then mounted on a cobalt-chromium frame. The newer generation valves—SAPIEN 3 and SAPIEN 3 Ultra Resilia—have outer sealing skirts made of polyethylene terephthalate (PET) to reduce paravalvular leaks [40,41]. Its balloon-expandable feature facilitates very precise annular deployment. Long-term data have demonstrated robust durability, low valve gradients, and low rates of SVD beyond 5 years [27,42].

#### 2.4.4. Medtronic Evolut

The Medtronic Evolut transcatheter aortic valve replacement (TAVR) system is a supra-annular self-expanding porcine bioprosthetic valve. It has a nitinol frame and a sealing skirt which helps it conform to the native annulus. The Evolut platform has undergone several iterations, including the Evolut R and Evolut PRO, which introduced features like re-capturability and an external pericardial wrap to better seal and reduce paravalvular leak [43]. The last addition in the Evolut family was the creation of larger frame cells, allowing easier engagement of the coronary ostia after valve implantation (Evolut FX+).

The Abbott Navitor valve is a self-expanding TAVR device. It is made from a nitinol frame with intra-annular leaflets and a sealing cuff to minimize paravalvular leak. Delivered via the FlexNav system, it allows for precise deliverability, especially in complex anatomies or smaller vessels. Similarly to Edwards SAPIEN and Medtronic Evolut valves, it offers excellent hemodynamics and advanced sealing technology, as well as large cells to allow coronary cannulation [44].

#### 2.4.5. Challenges/Management

While TAVR can offer a safe, feasible option for otherwise high-risk patients, it still faces numerous limitations. There are a number of anatomical considerations that are factored into the preoperative planning of a TAVR procedure, such as aortic annular diameter, degree and location of calcifications, height of the coronary ostia, and peripheral arterial disease. Not infrequently, patients may have anatomical features that are prohibitive for TAVR. Notable challenges of TAVRs are paravalvular leaks (PVLs), risk of stroke, need for a permanent pacemaker, and vascular complications. PVLs are most commonly due to incomplete expansion of the stent frame in proximity of significant calcium nodules [45,46]. Severe PVL is associated with increased mortality [45,46,47,48], and newer generation TAVR valves are designed with novel paravalvular sealing mechanisms [49,50].

Furthermore, similarly to all cardiac procedures, TAVR carries a risk of perioperative stroke. In a study looking at 11,957 patients who received TAVRs over ten years, the 30-day incidence rate of stroke was 3.0%, with 69% of those occurring within the first 48 h after TAVR. The incidence of stroke was 4.3% at 1 year and 7.8% at 5 years [51]. Strokes following TAVRs are commonly embolic, as more calcific native valves carry higher risks. Cerebral embolic protection devices are frequently used during TAVR procedures to mitigate these risks, although their efficacy has not been determined yet [52].

The combination of the proximity of the bundle to the annular level, and the various degrees of over-expansion of the TAVR valves, contributes to the conduction abnormalities frequently seen after THV implantation. Left bundle branch block, complete heart block, junctional rhythms, and other bradyarrhythmias are the most frequent causes of PPM implantation after TAVR. Despite improvement in valve technology and strategies of implantation, PPM rate remains higher for self-expandable (26%) compared to balloon-expandable valves (7%) [53].

Vascular complications are a known risk of TAVR due to large-bore arterial access, and may include bleeding, arterial dissection, perforation, and pseudoaneurysm. Despite improved sheath profiles and closure devices, major vascular complications still occur in 4–6% of cases and are associated with increased morbidity and mortality [54,55]. Risk factors include peripheral artery disease and small or calcified iliofemoral arteries. Pre-procedural imaging and access planning remain key to prevention.

The long-term durability of TAVR valves remains under investigation, especially as use expands to younger, lower-risk patients [56]. The NOTION trial, the only randomized study with 10-year follow-up, showed no differences in mortality, stroke, or MI between TAVR and SAVR, and found lower rates of structural valve deterioration (SVD) with TAVR; all patients received self-expanding [57]. The 5-year PARTNER 3 trial similarly found no significant differences in outcomes in low-risk patients treated with balloon-expandable valves, and 10-year data from PARTNER 2 showed comparable long-term outcomes in intermediate-risk patients [58,59]. While encouraging, broader and longer-term data are still needed.

#### 2.4.6. The Future of TAVR

The evolution of TAVR technology has led to exciting advancements in the management of aortic stenosis patients and improving outcomes across the board. The future of TAVRs foresees smaller delivery systems, a wider selection of sizes to cover a broader range of annular areas, markers to optimize implantation depth, options for coronary alignment, and a variety of deployment techniques to streamline their implantation. Moreover, as a result of the latest early-TAVR trial showing improved outcomes of TAVRs in asymptomatic patients with severe AS, it is possible that the current guidelines might change, identifying this cohort of patients as a potential target for early treatment [60].

Bicuspid aortic valves (BAV) present unique anatomical challenges for both surgical and transcatheter interventions, including elliptical annuli, asymmetric and bulky calcifications, and associated aortic pathologies, factors often complicating procedural planning and valve deployment [61]. Nevertheless, multiple large registries and meta-analyses demonstrate that TAVRs in BAV patients are both safe and feasible, with device success (~97%) and 1-year survival (≈91%) similar to tricuspid aortic valves; however, there are increased risk of paravalvular leak, peri-procedural stroke, and pacemaker rates [62]. Importantly, in low-risk bicuspid populations, TAVR has shown favorable outcomes, including lower 1-year mortality, supporting its expanding role beyond high-risk cohorts.

Not only will transcatheter devices become the first line of treatment for patients with aortic stenosis across all risk profiles, but TAVR technology will soon be adopted for the treatment of aortic valve insufficiency. To date, there are no FDA-approved TAVR devices for the treatment of aortic regurgitation (AR).

The JenaValve Trilogy TAVR is a new system currently being studied in the ALIGN-AR pivotal trial to assess the safety and efficacy of the device in treating high-risk patients with symptomatic, severe AR, with the results of the study anticipated in late 2025 [63].

Another exciting advancement is the use of Artificial Intelligence (AI) in TAVR procedures. AI systems could potentially improve patient selection, procedural planning, and post-implantation monitoring, thereby optimizing patient outcomes [64]. Computed tomography 3D reconstruction using AI, can more accurately predict optimal valve type and size when compared to transthoracic echocardiography [65]. Furthermore, machine-learning models can predict post-TAVR implantation transvalvular gradients with different valve sizes/types, thereby guiding the decision making during the preoperative planning phase [66].

Additionally, advancements in valve durability are critical as TAVR indications extend to younger and lower-risk populations with longer life expectancies. Ongoing research and long-term data collection will be essential to address these challenges and solidify TAVR’s role in the comprehensive management of aortic valve disease.

#### 2.4.7. TAVR Versus SAVR

The rate of TAVR has surged over the past decade, rivaling SAVR, not only in high-risk old patients, but also in younger individuals. A retrospective analysis of 9557 patients who underwent biological aortic valve replacement between 2013 and 2021 showed an increase in rates of patients < 65 receiving TAVR 7.1% in 2013 to 54.7% by 2021. Nonetheless, the study also showed TAVR was associated with higher 6-year mortality compared to SAVR (23.3% vs. 10.5%; hazard ratio, 2.27; 95% CI, 1.82–2.83; *p* < 0.001). Additionally, the 30-day rate of new pacemaker implantation was higher in the TAVR group (10.7% vs. 6.2%; *p* < 0.001) [67]. However, there were no significant differences between TAVR and SAVR in the 6-year cumulative incidence of stroke, heart failure hospitalizations, or reoperations. These findings underscore the necessity for longitudinal studies to evaluate long-term outcomes of TAVR versus SAVR in younger patient populations.

A subanalysis of the PARTNER trials, conducted between 2007 and 2011, evaluated stroke risk among patients undergoing TAVR versus SAVR. A propensity-matched analysis of 1204 patient pairs demonstrated that the 30-day stroke rate was similar between the two groups (5.1% for TAVR vs. 3.7% for SAVR; *p* = 0.09). However, the incidence of 30-day major stroke was significantly lower in the TAVR group compared to the SAVR group (3.9% vs. 2.2%; *p* = 0.018) [68]. Both groups exhibited a peak in stroke risk within the first post-procedure day, followed by a consistent low-level risk up to 48 months. Overall, these studies highlight the need for comprehensive patient risk evaluation to guide the complex decision of which AVR modality would be optimal.

#### 2.4.8. Failed Bioprosthesis Management with TAVR

As previously discussed, the currently available bioprostheses have limited durability of 10–15 years. Therefore, an increasingly important topic is the management of bioprosthesis failure. Traditionally, this is managed with re-operation, bioprosthesis explant, and redo SAVR. However, TAVRs provide new options in this arena such as Valve in Valve (ViV) procedures, which entails the deployment of a TAVR valve within an existing aortic bioprosthesis. To date, several non-randomized studies compared isolated redo SAVR vs. ViV TAVR. While operative mortality rates are similar, the observed-to-expected operative mortality in the redo SAVR group seems higher than ViV TAVR (1.2 vs. 0.32). Patients with SAVRs have higher rates of renal failure and blood transfusions [69]. More studies are ongoing, analyzing not only short-term outcomes of ViV interventions but also mid- to long-term results of these procedures, assessing hemodynamic profiles of the transcatheter valves, clinical implications, and durability of the prosthesis.

## 3. Discussion/Conclusions

The evolution of aortic valve replacement over the past several decades illustrates the rapid advancements in cardiovascular surgery and interventional cardiology. From the introduction of the Starr-Edwards valve in 1961 to the recent and transformative adoption of transcatheter aortic valve replacement, AVR development has expanded options for patients with aortic valve disease.

Surgical Aortic Valve Replacement (SAVR) remains the gold standard for younger patients, who benefit from the durability of mechanical prostheses. The development of bileaflet valves and innovations such as the On-X valve have improved hemodynamics and reduced the burden of anticoagulation, although lifelong anticoagulation and risk of thromboembolism remain an inherent limitation. Bioprosthetic valves, while eliminating the need for chronic anticoagulation, continue to face the challenge of structural valve deterioration (SVD). Advances in anti-calcification treatments and valve-in-valve (ViV) strategies are potential directions in this arena, in the effort of avoiding re-operation.

The rise in TAVR has redefined the treatment for aortic stenosis, especially for elderly or high-risk surgical patients. TAVR conveys lower procedural morbidity, shorter recovery times, and expands therapies to previously inoperable patients. Successive trials of PARTNER 1 through 3 and CoreValve low-risk have demonstrated efficacy across broader demographics and all risk profiles, including low-risk patients [37,38,39,42]. However, TAVR is not without its complications. Paravalvular leak, conduction disturbances requiring pacemaker implantation, and limited long-term valve durability continue to challenge its widespread adoption.

The increasing use of TAVR among patients under 65 years of age is an important trend. While TAVR offers short-term benefits, data suggest that SAVR may still provide superior long-term survival in this population. The observed higher incidence of pacemaker implantation and mortality in younger TAVR recipients highlights the critical need for careful patient selection, and a shared heart team approach is critical to optimize and individualize the therapy for patients with aortic valve disease, either surgical or transcatheter. Moreover, randomized control trials focusing on younger and lower-risk populations are essential to advance the indications for TAVR.

In summary, the future of AVR is rapidly evolving, highlighted by the plethora of treatment modalities developed over the years, with even more coming down the pipeline. Looking ahead, the next milestones in AVR should focus on individualization and precision. Promising efforts are underway to integrate the use of AI for procedural planning, imaging interpretation, risk modeling, and decision making. As AVR techniques continue to evolve, the goal remains the same: to provide safe, durable, and effective treatment for patients with aortic valve pathologies. Continued innovation, coupled with rigorous scientific inquiry, will guide the future of the field.

## Data Availability

No new data were created or analyzed in this study. Data sharing is not applicable to this article.
